# The expression of Eps15 homology domain 1 is negatively correlated with disease-free survival and overall survival of osteosarcoma patients

**DOI:** 10.1186/s13018-019-1137-6

**Published:** 2019-04-11

**Authors:** Hongwei Yu, Guofan Qu, Yuxue Wang, Wei Mai, Jun Jie Bao, Chunyu Song, Meng Yao

**Affiliations:** 1 Department of Orthopaedics, The Tumor Hospital Affiliated to Harbin Medical University, No.150, Haping Road, Xiangfang District, Harbin, 150040 Heilongjiang China; 2 Department of Orthopaedics, The Second Affiliated Hospital of Harbin Medical University, No. 246, Xuefu Road, Nangang District, Harbin, 150001 Heilongjiang China

**Keywords:** Osteosarcoma, Eps15 homology domain 1, Disease-free survival, Overall survival

## Abstract

**Background:**

Osteosarcoma was locally aggressive and frequently metastasizes to the lung. However, the etiology of osteosarcoma was unknown. Thus, exploring the mechanisms behind the occurrence of osteosarcoma was important for its prediction and prevention. To investigate the usefulness of mammalian Eps15 homology domain 1 (EHD1) as a prognostic marker for osteosarcoma, the expression of EHD1 in 57 osteosarcoma patients was measured using immunohistochemistry techniques and correlated with the clinicopathological features of patients.

**Methods:**

Correlations of EHD1 expression levels with clinicopathological features of patients were assessed using the Pearson *χ*^2^ test for categorical variables and the Student *t* test for continuous variables. Cumulative disease-free survival (DFS) curves and overall survival (OS) curves were plotted using the Kaplan–Meier method, and the relationship between each of the variables and survival was assessed by log-rank tests using univariate analysis. Subsequently, the parameters were tested using the multivariate Cox proportional hazards model, which was used to identify independent variables for predicting survival. EHD1 expression [*P* = 0.020; HR, 5.582; 95% confidence intervals (CI), 1.314–23.72] was an independent prognostic indicator of DFS in osteosarcoma patients; tumor size and EHD1 expression of osteosarcomas were independent prognostic indicators of OS in osteosarcoma patients.

**Results:**

EHD1 protein expression was a positive expression in examined tumor tissues. The median OS time of patients with high expression of EHD1 was 46.8 months (95% CI, 29.8–63.8 months), and the median OS time of patients with low expression of EHD1 was 58.8 months (95% CI, 31.6–86.0 months). The prognosis for patients with low expression of EHD1 in osteosarcomas was significantly better than that for patients with high expression of EHD1 (log-rank test, *P* = 0.019).

**Conclusion:**

The expression of EHD1 was negatively correlated with DFS and OS of osteosarcoma patients; therefore, the expression of EHD1 is a prognostic marker for prediction and prevention of osteosarcomas.

## Introduction

Osteosarcoma, a malignant tumor that frequently occurs in the distal femur, the proximal tibia, and the proximal humerus [1], is locally aggressive and frequently metastasizes to the lung [2]. A combination of surgery and chemotherapy results in the long-term survival of approximately 60–70% of osteosarcoma patients [3]. However, the etiology of osteosarcoma is unknown. Because of this unknown etiology, prevention and early diagnosis of osteosarcoma is difficult. Thus, exploring the mechanisms behind the occurrence of osteosarcoma is important for its prediction and prevention.

Mammalian Eps15 homology domain 1 (EHD1) is located in the chromosomal band 11q13 and is associated with lung, breast, head, neck, and small-cell lung cancer [4]. EHD1 plays an important role in the control of various cellular events by regulating various proteins [5, 6], including the β1 integrins [7]. The β1 integrins can bind to the extracellular matrix and stimulate the signaling pathways that influence the proliferation, apoptosis, cell spreading, migration, invasion, and metastasis of tumor cells [4]. In addition, high levels of EHD1 expression were associated with poor response to treatment in patients with cutaneous T cell lymphomas [8]. The studies were also reported that EHD1 was the best-studied member of the four highly homologous mammalian proteins (EHD1–4) which regulated the endocytic recycling of membrane and associated cell surface receptors [9, 10]. These studies suggest that EHD1 plays a role in cancer invasion and metastasis.

The present study aimed to investigate the usefulness of EHD1 as a prognostic marker for osteosarcoma. The expression of EHD1 in tumor and normal tissues collected from 57 osteosarcoma patients was measured using immunohistochemistry techniques, and correlations with the clinicopathological features of patients were sought.

## Methods

### Patients

To assess the prognostic capacity of EHD1 for osteosarcomas, formalin-fixed, paraffin-embedded (FFPE) SCLC tumor tissues were collected from 57 osteosarcoma patients who underwent surgery between January 2011 and September 2015. Inclusion criteria were patients recorded to have osteosarcoma by pathology reports and included cases from stage I to stage III. All cases, representing a spectrum of osteosarcomas, were retrieved from patients attending Harbin Medical University Cancer Hospital, Harbin, China. Exclusion criteria were having stage IV of disease, or a history of other cancers. For each patient, each tissue specimen type was resected during a single surgical procedure. Primary cancers were evaluated in accordance with the seventh edition of the American Joint Committee on Cancer (AJCC) staging system (TNM). All patients were followed up until their death or the study end date (January 21, 2014). The median follow-up time for survivors was 46.8 months (range 34.9–58.7 months). The study was approved by the Ethical Committee of Harbin Medical University. Informed consent was obtained from all patients. All investigators involved in the study, apart from the study statistician, were blinded to patient outcomes throughout all laboratory analyses.

### Immunohistochemistry

The expression of EHD1 in FFPE sections was analyzed by immunohistochemistry. The tissue sections were first dried at 70 °C for 3 h. After deparaffinization and hydration, sections were washed in phosphate-buffered saline (PBS, 3 × 3 min). Endogenous peroxidase was quenched with 3% H_2_O_2_ for 15 min. After first being washed with distilled water, sections were washed in PBS (3 × 5 min). Antigen retrieval was performed in citrate buffer (pH 6.0). Each section was treated with 300–500 ml EHD1 rabbit polyclonal antibody solution (Abcam, Cambridge, UK, ab75886, diluted at 1:200) overnight at 4 °C. The sections were incubated with peroxidase-conjugated streptavidin for 30 min, and the reaction products were visualized with diaminobenzidine as a chromogen and counterstained with commercial hematoxylin. The percentage of positive cells was determined by counting 500 cells in five randomly selected fields per section. IHC staining was scored based on intensity as follows: 0 (no staining), 1 (weak staining = light yellow), 2 (moderate staining = yellow brown), and 3 (strong staining = brown). The percentage (0–100%) of the extent of reactivity was scored as follows: 0 (no positive tumor cells), 1 (fewer than 10% positive tumor cells), 2 (10–50% positive tumor cells), and 3 (greater than 50% positive tumor cells). Next, the cytoplasmic expression score was obtained by multiplying the intensity and reactivity rate values. Scores of < 4 were classified as low expression, and the remainders were classified as high expression. Two blinded, independent observers interpreted all slides.

### Statistical analysis

All analyses were performed using statistical software (SPSS 19.0 for Windows; SPSS, Inc., Chicago, IL, USA). Differences were considered statistically significant when *P* < 0.05. Correlation of EHD1 expression levels with clinicopathological features of patients was assessed using the Pearson *χ*^2^ test for categorical variables and the Student *t* test for continuous variables. Disease-free survival (DFS) was calculated from the date of surgery resection to the date of last follow-up or relapse. Cumulative DFS curves and overall survival (OS) curves were plotted using the Kaplan–Meier method, and the relationship between each of the variables and survival was assessed by a log-rank test using univariate analysis. Covariates with *P* ≤ 0.15 in the univariate analyses were included in multivariate analyses. The parameters were then tested using the multivariate Cox proportional hazards model, which was performed to identify independent variables for predicting survival. Risk ratios and their 95% confidence intervals (CIs) were recorded for each factor.

## Results

### EHD1 protein expression in tumor tissues

**Fig. 1:**
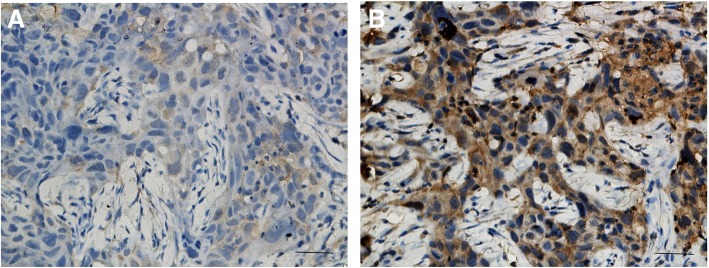
Immunohistochemical staining of EHD1 in FFPE tissue samples (× 40). **a** EHD1 low expression. **b** EHD1 high expression

EHD1 protein expression was a positive expression in examined tumor tissues (Fig. 1). The cytoplasmic staining patterns observed for EHD1 were consistent with data from our previous studies.

### Association of EHD1 protein expression and clinicopathological features

Correlation of EHD1 expression with the clinicopathological features of patients, including age, gender, tumor histology, tumor size, and AJCC stage in SCLC patients was assessed (Table 1). High expression of EHD1 in tumor tissues was positively correlated only with the disease type (*P* = 0.025). No such significant correlations between EHD1 and other clinicopathological features were found in this study.

**Table 1:** The correlation between clinicopathological features and EHD1 expression

	EHD1 expression		
	Low (*n* = 28)	High (*n* = 29)	*P*
Gender			0.707
Male	17 (60.7%)	19 (65.5%)	
Female	11 (39.3%)	10 (34.5%)	
Age	23.0 ± 16.0	24.5 ± 14.8	0.705
Disease type			0.025*
Chondroblastomas	8 (28.6%)	1 (3.4%)	
Other	20 (71.4%)	28 (96.6%)	
Tumor size			0.872
≥ 5 cm	17 (60.7%)	17 (58.6%)	
< 5 cm	11 (39.3%)	12 (41.4%)	
Stage			0.206
I	0 (0%)	2 (6.9%)	
II	27 (96.4%)	24 (82.8%)	
III	1 (3.6%)	3 (10.3%)	

[0 (negative) ≤ score ≤ 1+] and [2+ ≤ score ≤ 3+] represent low negative and strong positive staining of EHD1, respectively. * *P* < 0.05

### Univariate and multivariate Cox regression analysis of potential prognostic indicators of DFS in osteosarcoma patients

No significant correlations between clinicopathological features and DFS, based on univariate Cox regression models, were found in this study. Multivariate Cox proportional hazards model analysis of the same set of patient data showed that EHD1 expression (*P* = 0.020; HR, 5.582; 95% CI, 1.314–23.72) was independent of prognostic indicators for DFS in osteosarcoma patients (Table 2).

**Table 2:** EHD1 expression in tumor tissues as an independent prognostic factor for DFS in osteosarcoma patients

	Univariate^a^		Multivariate^b^	
	HR (95% CI)	*P*	HR (95% CI)	*P*
Gender (male vs female)	1.363 (0.505–3.676)	0.541	1.581 (0.549–4.552)	0.396
Age	0.974 (0.939–1.01)	0.157	0.985 (0.912–1.065)	0.704
Disease type (chondroblastomas vs other)	1.468 (0.421–5.122)	0.547	4.336 (0.76–24.738)	0.099
Tumor size (≥ 5 cm vs < 5 cm)	1.306 (0.504–3.383)	0.582	1.881 (0.616–5.748)	0.267
EHD1 expression (high vs low)	2.771 (0.981–7.824)	0.054	5.582 (1.314–23.72)	0.020

*CI* confidence interval, *HR* hazard ratio, *DFS* disease-free survival

^a^Variables were adopted for their prognostic significance (*P* < 0.05) in univariate analysis using forward, stepwise selection (forward likelihood ratio)

^b^A Cox proportional hazards regression model was used for multivariate analysis

### Univariate and multivariate Cox regression analysis of potential prognostic indicators of OS in osteosarcoma patients

Univariate Cox regression model analysis showed that age (*P* = 0.015; HR, 0.945; 95% CI, 0.903–0.989), tumor size (*P* = 0.042; HR, 3.155; 95% CI, 1.045–9.525), and EHD1 expression (*P* = 0.026; HR, 3.202; 95% CI, 1.149–8.92) were independent prognostic indicators of OS in osteosarcoma patients (Table 3). Analysis of the same set of patient data using a multivariate Cox proportional hazards model showed that tumor size (*P* = 0.008; HR, 5.854; 95% CI, 1.587–21.597) and EHD1 expression (*P* = 0.007; HR, 6.372; 95% CI, 1.645–24.676) were independent prognostic indicators of OS in osteosarcoma patients (Table 3).

**Table 3:** EHD1 expression in tumor tissues as an independent prognostic factor for OS in osteosarcoma patients

	Univariate^a^		Multivariate^b^	
	HR (95% CI)	*P*	HR (95% CI)	*P*
Gender (male vs female)	0.927 (0.368–2.335)	0.872	1.323 (0.468–3.743)	0.597
Age	0.945 (0.903–0.989)	0.015	0.988 (0.887–1.101)	0.833
Disease type (chondroblastomas vs other)	0.876 (0.201–3.829)	0.861	2.147 (0.346–13.314)	0.412
Tumor size (≥ 5 cm vs < 5 cm)	3.155 (1.045–9.525)	0.042	5.854 (1.587–21.597)	0.008
EHD1 expression (high vs low)	3.202 (1.149–8.92)	0.026	6.372 (1.645–24.676)	0.007

*CI* confidence interval, *HR* hazard ratio, *DFS* disease-free survival

^a^Variables were adopted for their prognostic significance (*P* < 0.05) in univariate analysis using forward, stepwise selection (forward likelihood ratio)

^b^A Cox proportional hazards regression model was used for multivariate analysis

### Kaplan–Meier survival curve analysis of DFS in osteosarcoma patients

**Fig. 2:**
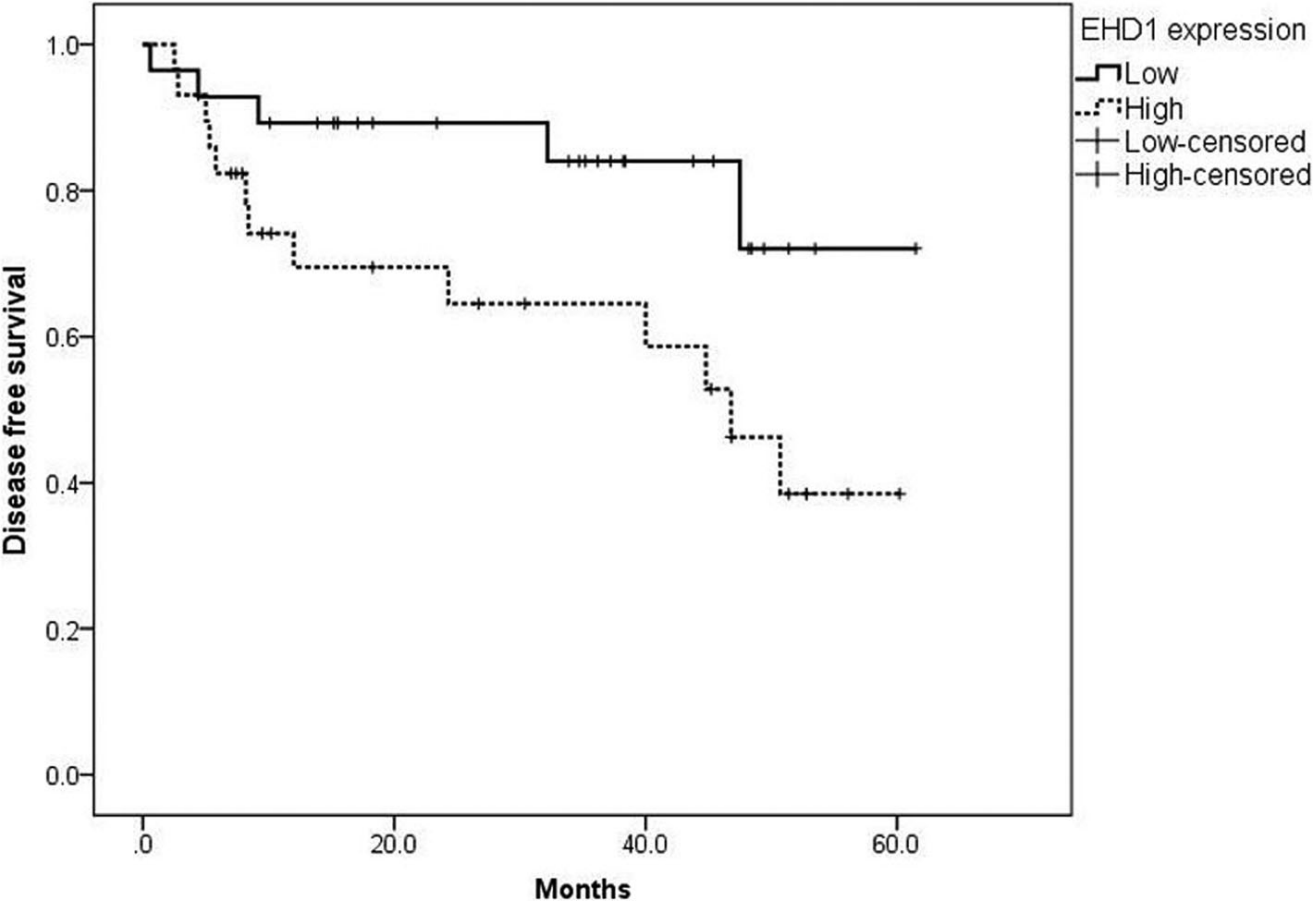
Kaplan–Meier analysis for disease-free survival (DFS) based on EHD1 expression status in osteosarcoma patients

Kaplan–Meier survival curves stratified for EHD1 expression are shown in Fig. 2. The median DFS time in EHD1 high-expression patients was 46.8 months (95% CI, 34.9–58.7 months). The prognosis for patients who had low expression of EHD1 in osteosarcomas was significantly better (log-rank test, *P* = 0.045) compared with patients who had high expression of EHD1.

### Kaplan–Meier survival curve analysis of OS in osteosarcoma patients

**Fig. 3:**
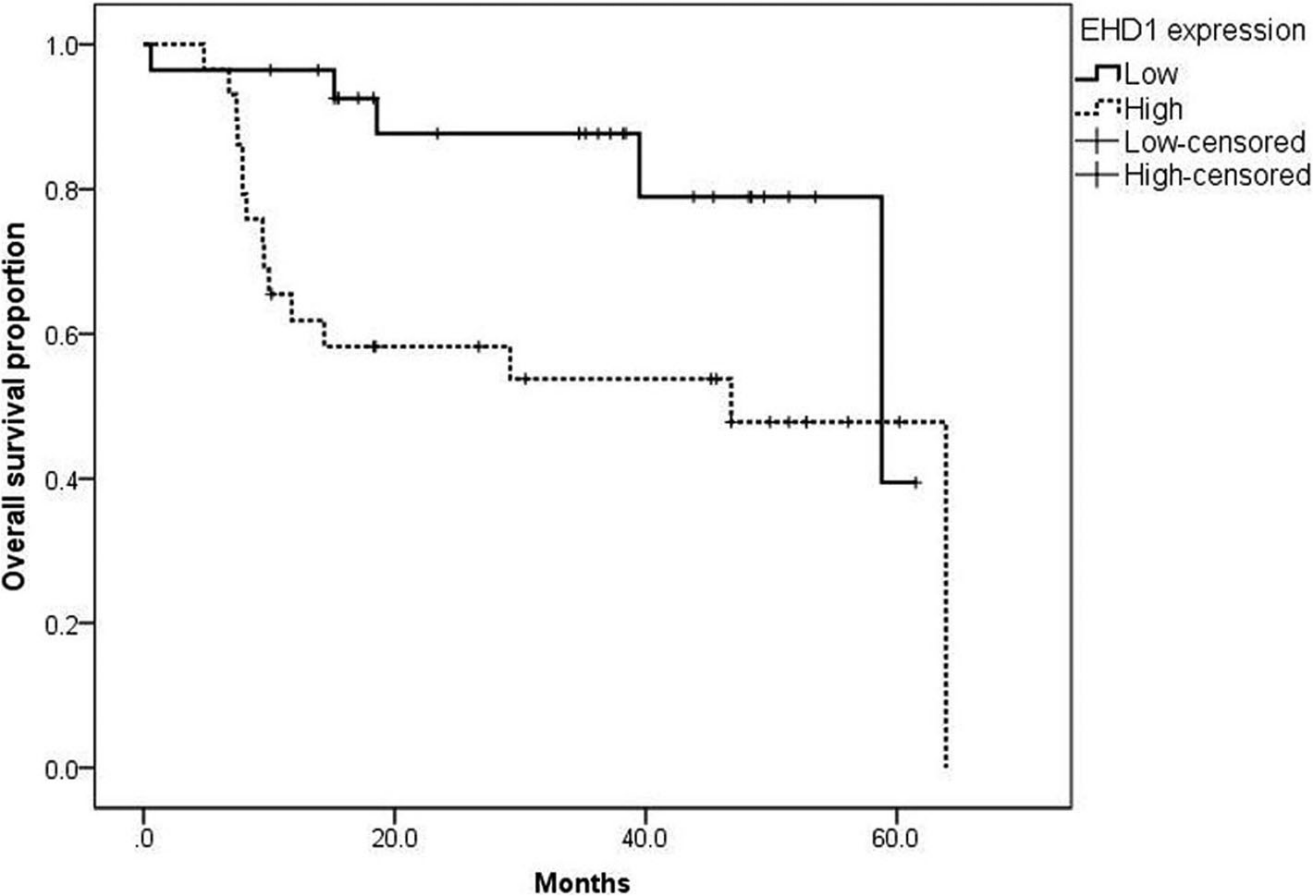
Kaplan–Meier analysis for overall survival (OS) based on EHD1 expression status in osteosarcoma patients

Kaplan–Meier survival curves stratified for EHD1 expression are shown in Fig. 3. The median OS time for patients with high expression of EHD1 was 46.8 months (95% CI, 29.8–63.8 months), and the median OS time for patients with low expression of EHD1 was 58.8 months (95% CI, 31.6–86.0 months). The prognosis for patients with low expression of EHD1 in osteosarcomas was significantly better (log-rank test, *P* = 0.019) compared with patients with high expression of EHD1.

## Discussion

Our results revealed that EHD1 expression was an independent prognostic indicator for osteosarcomas, which was similar to results found in previous studies reporting the prognostic value of EHD1 expression in both non-small-cell and small-cell lung cancer [4]. High levels of EHD1 expression have also been associated with poor response to treatment in patients with cutaneous T cell lymphomas [8]. EHD1 is the best-studied member of the four highly homologous mammalian proteins (EHD1–4) which regulate endocytic recycling of membrane and associated cell surface receptors [9, 10]. EHD proteins appear to be involved in critical nodes in the endocytic sorting/recycling process [4]. In other reports, the reports suggested that EHD1 participated in regulating the rate of recycling endocytic compartments traveling to the cell surface via trafficking as part of the slow recycling pathway [11, 12], and the decreased expression of EHD1 enhanced the metastatic ability of well-differentiated pancreatic endocrine neoplasms [13, 14]. EHD1 can be found in exosome secreted by prostate cancer cells [15, 16]. Increased EHD1 expression in lesions associated with cutaneous T cell lymphomas indicated a poor response to treatment [8, 17]. Considering all of these observations, the expression of EHD1 could be considered as a prognostic marker for osteosarcoma prediction and prevention.

In addition, tumor size and EHD1 expression were found to be independent prognostic indicators for osteosarcomas. The median DFS time in patients who showed high expression of EHD1 was 46.8 months, and the prognosis for patients with low expression of EHD1 in osteosarcomas was significantly better than those with high expression of EHD1. The median OS time in patients with high expression of EHD1 was 46.8 months, and the median OS time in patients with low expression of EHD1 was 58.8 months. The prognosis for patients with low expression of EHD1 in osteosarcomas was significantly better than those with high expression of EHD1. A previous study showed that EHD1 expression is associated with a poor response to chemotherapy in cutaneous T cell lymphoma patients, which is consistent with our results [4]. The expression of EHD1 was negatively correlated with DFS and OS in osteosarcoma patients, and thus, the expression of EHD1 could be considered as a prognostic marker for osteosarcoma prediction and prevention. Notwithstanding this study’s limitation, in that it had such a small sample size, the identification of the prognostic potential of EHD1 expression warrants further study in larger cohorts to validate the effects found in osteosarcoma patients.

## Conclusion

EHD1 expression was found to be an independent prognostic indicator of OS in osteosarcoma patients. The prognosis for patients with low expression of EHD1 in osteosarcomas was significantly better than patients with high expression of EHD1. The expression of EHD1 was negatively correlated with DFS and OS of osteosarcoma patients, and thus, the expression of EHD1 could be considered as a prognostic marker for the prediction and prevention of osteosarcoma.
